# Information resources to aid parental decision-making on when to seek medical care for their acutely sick child: a narrative systematic review

**DOI:** 10.1136/bmjopen-2015-008280

**Published:** 2015-12-16

**Authors:** Sarah Neill, Damian Roland, Caroline HD Jones, Matthew Thompson, Monica Lakhanpaul

**Affiliations:** 1 School of Health, University of Northampton, Northampton, UK; 2 Sapphire Group, Health Sciences, University of Leicester, Leicester, UK; 3 Paediatric Emergency Medicine Leicester Academic (PEMLA) Group, Leicester Hospitals, Leicester, UK; 4 Nuffield Department of Primary Care Health Sciences, University of Oxford, Oxford, UK; 5 Department of Family Medicine, University of Washington, Seattle, USA; 6 Department of Population, Policy and Practice, Institute of Child Health, University College London, London, UK

**Keywords:** PUBLIC HEALTH, PAEDIATRICS

## Abstract

**Objective:**

To identify the effectiveness of information resources to help parents decide when to seek medical care for an acutely sick child under 5 years of age, including the identification of factors influencing effectiveness, by systematically reviewing the literature.

**Methods:**

5 databases and 5 websites were systematically searched using a combination of terms on children, parents, education, acute childhood illness. A narrative approach, assessing quality via the Mixed Methods Appraisal Tool, was used due to non-comparable research designs.

**Results:**

22 studies met the inclusion criteria: 9 randomised control trials, 8 non-randomised intervention studies, 2 qualitative descriptive studies, 2 qualitative studies and 1 mixed method study. Consultation frequency (15 studies), knowledge (9 studies), anxiety/reassurance (7 studies), confidence (4 studies) satisfaction (4 studies) and antibiotic prescription (4 studies) were used as measures of effectiveness. Quality of the studies was variable but themes supported information needing to be relevant and comprehensive to enable parents to manage an episode of minor illness Interventions addressing a range of symptoms along with assessment and management of childhood illness, appeared to have the greatest impact on the reported measures. The majority of interventions had limited impact on consultation frequencies, No conclusive evidence can be drawn from studies measuring other outcomes.

**Conclusions:**

Findings confirm that information needs to be relevant and comprehensive to enable parents to manage an episode of minor illness. Incomplete information leaves parents still needing to seek help and irrelevant information appears to reduce parents’ trust in the intervention. Interventions are more likely to be effective if they are also delivered in non-stressful environments such as the home and are coproduced with parents.

**Strengths and limitations of this study**

- This is the first review of the outcome of information resources which aid parental decision-making utilising systematic search and quality assessment criteria.
- The strengths of this review lies in its inclusiveness. Using an integrative narrative approach enabled us to identify influences on effectiveness across a wider range of studies and topics than would have been possible with a single study type or topic focused review.
- The findings are limited by the quality of the studies and not being able to control for the impact of different healthcare delivery systems.

## Background

Acute illness is a universal experience for children and families and represents the most common type of illness in childhood, particularly in 0–5-year-olds. Acute illness includes short-term illnesses, predominantly infections such as coughs, colds, diarrhoea, vomiting and ear infections. Home management is often supported by consultations in primary care, where children under 5 years old constitute 40% of general practitioner (GP) workload,^1^ with most consultations for acute illness.^2^ ^3^ Under 1-year olds are seen more often than all other age groups other than the over 75s^2^ and urgent care and emergency department service use by young children appears to be rising.^4–6^

Parents’ anxiety about acute childhood illness leads them to seek information to help them decide whether or not to seek help from a healthcare professional.^7–11^ A wide range of information is available for families, such as written leaflets or via websites much of which is either unknown to parents^5^ ^7^ or does not seem to be making any impact on service use when children are acutely sick at home.^11–14^ The increase in consultation rates for non-urgent care^4–6^ suggests more effective information sources are needed.

We aimed to systematically review the literature to identify the effectiveness of information resources to help parents decide when to seek medical care for an acutely sick child under 5 years of age, including the identification of factors influencing effectiveness.

Our research questions were:

- What measures of effectiveness have been used to evaluate such interventions?
- How effective are existing interventions in helping parents know when to seek help for an acutely sick child at home?
- What factors influence effectiveness of information provision to help parents know when to seek help for an acutely sick child at home?

## Methods

### Search strategy

We systematically searched five electronic databases (MEDLINE, CINAHL, PsycNET, ASSIA Web of Knowledge) and five websites (Centre for Review and Dissemination York, National Institute for Health and Care Excellence, Health Technology Assessment programme, NHS Evidence and the Cochrane Library) using a combination of terms on children, parents/carers, education, acute childhood illness (see online supplementary appendix 1). We scanned reference lists of key articles, and attempted to contact authors when further information was required to determine eligibility and inform quality assessment.

### Selection criteria

Studies which met all the following criteria were included:

1. Studies which included children from 0 to 14 years with research participants being their parents or caregivers. Initial pilot searches aimed solely at children under 5 years yielded minimal results.
2. An educational intervention on acute childhood illness was provided to parents/caregivers in any form (written, visual, verbal or electronic) designed to help with decision-making about whether or not to seek medical help.
3. The study was conducted in primary care, emergency departments, ambulatory settings or in the home, in high income countries as defined by Organisation for Economic Co-operation and Development (OECD). We included all study types.

Studies were excluded if they focused on chronically ill children, hospital inpatient settings or educational interventions designed for health professionals. We limited our search to papers published in the English language, between January 1990 and June 2014 (inclusive). The decision to search from 1990 was taken pragmatically as health services have evolved considerably since the latter half of the 20th century. We did not exclude studies on the basis of quality alone but have noted the quality of studies when discussing their impact. To have excluded low quality studies would have reduced the comprehensiveness of the review, especially given the likely heterogeneity of study design.

The titles and abstracts of studies identified in the search were retrieved and assessed by one reviewer who excluded those that were clearly not relevant. The full text of remaining studies was assessed for inclusion by two reviewers; discrepancies were resolved by discussion between all authors. Reasons for exclusion were recorded (see online supplementary appendix 2).

### Data extraction and quality assessment

Data from included studies were extracted by one reviewer and checked by a second reviewer. All studies which met the inclusion criteria were included regardless of quality, which was assessed independently by two other reviewers using the Mixed Methods Appraisal Tool (MMAT).^15^ This gives a rating between zero stars (lowest quality) and 4 stars (****, highest quality).

### Evidence synthesis: synthesising qualitative and quantitative research

Narrative review was used to summarise and explain findings across studies.^16^ ^17^ Meta-analysis was inappropriate due to non-comparable research designs.

## Results

**Figure 1:**
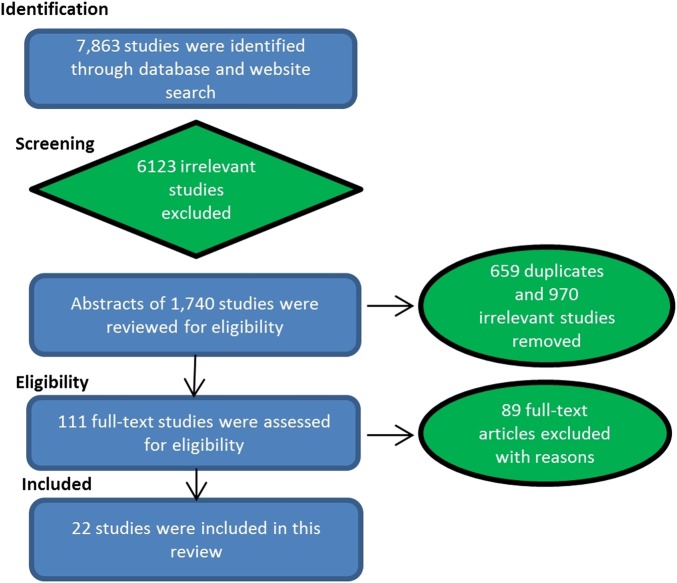
Flow of information through the phases of the selection process (using PRISMA Flow Diagram structure (Moher *et al*., 2009)). Refer to appendix 2 for reasons for exclusion.

The search identified 7863 studies, of which 22 were included (figure 1). Table 1 shows the characteristics of included studies of which there were nine randomised controlled trials, eight non-randomised intervention studies, two qualitative descriptive studies, two qualitative studies and one mixed method study. Thirteen were conducted in the USA, six in the UK, two in Canada and one in Denmark. Parents/caregivers of children aged 0–14 years were included across all studies, with 12 studies limiting inclusion to parents of children under the age of 6 years. Studies were conducted in primary care (9), emergency department/hospital (7), child health clinics (3) and children's health centres (3).

**Table 1:** Characteristics and quality assessment of studies included

Author(s)/Date	Setting	Aim	Design	Sample	Intervention	Main outcomes	Quality assessment*
Qualitative studies							
Kai 1994^32^	Health visitor and general practitioner baby clinics (UK)	To explore disadvantaged parents’ perceptions and use of the Baby Check booklet	Qualitative interview and records of consultations	Parents of 34 babies <6 months attending weekly baby clinic in GP in disadvantaged area	Parents were given a copy of Baby Check. Unstructured 30–90-min interviews with parents until baby was 6 months	Perceptions, use of the booklet and consultations for illness among disadvantage parents	**
Krantz 2001^38^	Parent Resource Centre. Children's Hospital Ontario (Canada)	To describe the development of, and pilot, a fever anticipatory guidance tool for parents	Qualitative interview	15 first-time parents with children aged 2 months to 4 years from inner city Parent Resource Centre	The Fever Anticipatory Guidance Tool	Views on, and use of, the booklet	*
Randomised controlled trials							
Baker *et al* 2009^18^	ED (USA)	Effect of a brief educational video during ED visit for minor febrile illnesses	RCT	280 parents of children aged 3 months to 3 years presenting to with febrile illness	*Intervention:* 11-min video on home management of fever. *Control:* 8-min video on home and automobile safety	Knowledge, attitudes, and return ED visits for minor febrile illnesses within 2 years	***
Broome *et al* 2003^19^	6 clinics in 6 states (USA)	Effect of a structured education programme on parents’/grandparents’ knowledge, confidence, and satisfaction in assessing and managing a child's fever	RCT	216 children from 3/12 to 6 years of age and their parents/grandparents. 183 followed up at 3 months and 145 at 6 months	*Intervention 1:* video and brochure on childhood fever in clinic; *Intervention 2:* brochure and video in clinic, plus health professional reinforced content and answered parents’ questions during consultation; *Control: ‘*usual’ care	Knowledge, confidence, and satisfaction in assessing and managing child’s fever at 48 h, 1, 3, and 6 months postintervention	*
Chande *et al* 1996^20^	Urban paediatric ED (USA)	Effect of educational intervention on common childhood illness on ED visits	RCT	130 parents of children with minor illnesses in ED	*Intervention:* 10-min video on paediatric healthcare issues plus information booklet on common paediatric ailments *Control:* standard ED discharge instructions	Return visits to ED over 6 months	*
Francis *et al* 2009^25^	General practice (UK)	Effect of interactive booklet on respiratory tract infections on reconsultation for same illness episode, antibiotic use, future consultation intentions, and parental satisfaction	Cluster RCT	61 practices in Wales and England. 558 parents of children (6 months to 14 years) with a respiratory tract infection	*Intervention:* Eight page booklet on childhood respiratory tract infections within consultations and as a take home *resource.* *Control: ‘*usual’ consultation	Reconsultation within 2 weeks, antibiotic prescribing and consumption, future consultation intentions, parent satisfaction and usefulness of information received, reassurance and enablement	****
Hansen 1990^26^	General practice (Denmark)	Effect of booklet on families’ minor illness-behaviour for children <8 years	RCT	100 young families with min. one child <8 years in one practice	*Intervention:* Booklet on common childhood problems, presented by GP. Parent recorded illnesses. *Control*: Unclear. ?‘usual care’ plus diary completion	Consultation frequency and anxiety over 6 months	**
McCarthy *et al* 1990^23^	US Private practice and primary care centre	Effect of Acute Illness Observation Scales (AIOS) on mother's judgements about acute illness in children under 24 months	RCT	369 mothers with 2-week-old baby	*Intervention:* AIOS film plus fever scenario scoring. Film shown again at 6 and 15 months. AIOS used to score illness prior to and with doctor during consultation. *Control:* Routine advice about fever. Illness scored on 3-point scale	Reliability, specificity and sensitivity of mother's judgements compared to clinician assessment from 2 weeks of age, for 32 months	*
Robbins *et al* 2003^12^	Primary care (UK)	Effect of home visit and infant minor illness booklet on parent's illness management and consultation rates	RCT	Single GP practice: 103 parents of babies born in 6-month birth cohort	*Intervention*: Postal booklet on common childhood illnesses. Research nurse visit when baby 6 weeks old. *Control:* Routine health visiting service	Confidence, knowledge, home care activities and desire to contact professionals. Prescription and consultation rates tracked for 6 months	***
Thomson *et al* 1999^33^	General Practice (UK)	Effect of Baby Check, an illness scoring system for babies ≤6/12, on parents’ use of health services for their baby	RCT	997 mothers with new babies	*Intervention:* Baby Check plus an accident prevention leaflet *Control:* accident prevention leaflet alone	Consultation behaviour tracked for 6 months	***
Usherwood 1991^35^	General practice (UK)	Effect of a children's symptom booklet on GP consultations	RCT	419 households with 634 children born 1975 to 1984 registered with one practice	*Intervention:* Postal booklet on cough, fever, sore throat, diarrhoea and vomiting *Control:* No intervention. Baseline data gathered for 2 months prior to intervention	Consultation rates for 12 months postintervention	*
Non-randomised trials							
Herman and Jackson 2010^29^	Head Start agencies (USA)	Effect of educational intervention on health utilisation for acute illness in children ≤5 years	Cohort study (prospective)	9240 parents with one child enrolled in Head Start 7281 completed the training 581 tracked annually for 2 years	Health training programmes using reference guide ‘What to Do When Your Child Gets Sick’ by Mayer and Kuklierus (2007) in 55 Head Start agencies in 35 states. Tracked for 3 months, trained in 4th month, follow-up for 6 months. Annual visits for 581 parents	ED and primary care consultation rates for 3-year period	***
Isaacman *et al* 1992^27^	Paediatric ED (USA)	Effect of two standardised simplified discharge instructions on parents information recall	CT (Non-randomised control)	197 parents of children discharged with otitis media (OM)	*Intervention 1:* standardised verbal discharge information on OM from HCPs in ED *Intervention 2:* as above+typewritten information from health professionals in ED.*Control:* ‘usual’ discharge information	Knowledge and management of OM before leaving ED, at 24 and 72 h postintervention Return visits to ED and parent reported physician contact within 72 h	**
Kelly *et al* 1996^36^	Private paediatrician's office, 4 Primary care centres (USA)	Effect of educational intervention on knowledge and management of fever	Pretest post-test cohort study	86 caretakers of children 2 months to 5 years presenting for routine healthcare or acute minor illness 50 follow-up interviews	Printed fever management sheet at end of initial interview Identified knowledge deficits addressed	Questionnaire on fever knowledge and management before and 2–4 weeks after intervention	**
O’Neill Murphy *et al* 2001^30^	Urban ED Children's Hospital of Philadelphia (USA)	Effects of educational programme on parents’ anxiety about fever, home management and consultation behaviour	Quasi-experimental, pretest post-test pilot study	87 parents with children aged 3 months to 5 years with fever >38.4	*Intervention:* Interactive Fever programme *Control:* Standard Fever Education Programme	Anxiety, consultation behaviour, home management before and after HCP consultation, 2 and 8 weeks after the intervention	*
Rosenberg and Pless 1993^21^	Montreal Children's hospital ED (Canada)	Effect of ED-based parent education on future ED visit rates	Non-randomised CT	300 parents of children >6 months in ED	*Intervention:* educational pamphlet on common childhood illness plus video in waiting room. *Control: ‘*usual’ care. (Sequential recruitment to intervention then control)	Consultation behaviour 4 and 12 months postintervention	
Steelman *et al* 1999^22^	Military Paediatric Clinic (USA)	Effect of educational intervention on parent's childhood fever knowledge and consultation rates	Pretest post-test CT	93 parents attending 2, 4, and 6 month well-infant visits	*Intervention:* standardised slide presentation on well-infant care+10 min presentation on fever and mail out at 1 and 3 months *Control*: standardised slide presentation on well-infant care	Knowledge of fever, clinic and ED usage at enrolment, 2 and 4 months postintervention	
Wassmer and Hanlon 1999^28^	Worcester Royal Infirmary DGH (UK)	Effect of information for parents on febrile convulsions on parent's knowledge	Non-Randomised CT	Intervention: 50 parents of children with 1st febrile convulsion May to Dec 1996. Control: 50 parents of children at community health clinic with no febrile convulsion	*Intervention:* verbal and written information on febrile convulsions during consultation *Control:* no information provided. Assume ‘usual care’	Parental knowledge of febrile convulsion 1 year postintervention	
Yoffe *et al* 2011^34^	Primary care clinic (USA)	Effect of parent-focused educational intervention on non-urgent ED visits	Realistic evaluation	Parents of all children ≤10 years attending 3 primary care clinics Number receiving the booklet was not provided	*Intervention:* booklet on common childhood illness to the parents with children registered with one primary care clinic *Control:* Parents of children registered with two other clinics not receiving the booklet	ED consultation rates Nov 2007 to Apr 2009	
Qualitative descriptive studies							
Thornton *et al* 1991^24^	Conducted in the home (UK)	Use of Baby Check (BC), an illness scoring system for babies ≤6/12, by mothers at home	Two field trails	Study A: 104 mothers of term babies, randomly selected from the birth register Study B: 70 mothers of term babies born on selected days	*Study A:* Mothers used BC daily for a week and recorded contacts with HCPs. Research nurse visit to grade mother's competence in booklet use *Study B:* Mothers used BC when wanted to until baby was 6 months. Research nurse visit when babies 8 and 16 weeks. Questionnaire about BC at 6 months	Views and use of the booklet	****
Anhang *et al* 2013^37^	Two Children's EDs (USA)	Usability and safety of a web-based decision support tool for parents of children with flu-like illnesses	Pilot feasibility study	294 parents/carers of children ≤18 years who had presented to an emergency department for an influenza-like illness	*Intervention:* Strategy for Off-site Rapid Triage (SORT) for Kids tool web-based parent survey and severity scoring tool	Caregiver ratings of usability of tool, sensitivity and specificity of SORT for Kids for identifying children needing ED	*
Mixed methods studies							
Stockwell *et al* 2010^31^	Early Head Start Agency at Columbia University (USA)	Pilot evaluation of a community-based, culturally competent health literacy intervention on care of URI, with Latino Early Head Start parents	Pretest post-test pilot evaluation	11 parents of children 6 months to 3 years in full evaluation 17 in interviews and 33 postclass evaluations	Three education modules delivered in children's centre	Parental knowledge, attitudes and care of URI before and 2 weeks after final module using Knowledge, Attitude, Practices instrument	**

*Quality assessment rating, between zero stars (lowest quality) and 4 stars (****, highest quality).

DGH, District General Hospital; ED, emergency department; GP, general practitioner; RCT/CT, randomised controlled trial/controlled trial; URI, upper respiratory infection.

Interventions involved written information in all but one study, which used video alone.^18^ Written information was augmented by video/slide presentations,^19–23^ home visits,^12^ ^24^ reinforcement within consultations^19^ ^23^ ^25–28^ or was part of a structured educational programme.^29–31^ Three separate studies reported on the same ‘Baby Check’ intervention in different settings/populations.^24^ ^32^ ^33^

Quality of included studies is summarised in table 1, and detailed in online supplementary appendix 3. Only two studies were given the highest quality score, with many being given low scores, often due to insufficient reporting of methods.

### Measures of effectiveness

The most frequently used measures of effectiveness were: consultation frequency (15 studies), parent knowledge (9 studies), parent anxiety/reassurance (7 studies), parent satisfaction (4 studies), parent confidence and clinician antibiotic prescribing (both 4 studies).

#### Consultation frequency

Six of the 15 studies which measured this outcome showed a significant reduction in either actual consultation rates or intention to consult in the future (see table 2). Three of these studies evaluated effects on consultation rates over a longer (1–3-year) period postintervention and found persistence of effect.^29^ ^34^ ^35^ (2 low and 1 high quality). One study (low quality) showed a reduction in home visits but with an increase in out-of-hours visits.^35^ The eight remaining studies on consultant frequency showed no difference on consultation rates with the specified intervention.

**Table 2:** Effectiveness of interventions on consultation rate

Authors (date)	Consultation rate (significant results in bold)	Quality
Anhang *et al* 2013^37^	The algorithm correctly classified 93% of paediatric patients with influenza-like illness who made necessary ED visits and all children who made a second ED visit for influenza-like illness within the subsequent week	*
Baker *et al* 2009^18^	No difference in reattendance to ED. p=0.46 95% CI −0.06 to 0.16	***
Chande *et al* 1996^20^	No difference in contact with primary care physician (p=0.37) or return visits to ED (p=0.68)	*
Francis *et al* 2009^25^	Non-significant reduction in reconsultation in first 2 weeks p=0.29 95% CI −2.7 to 9.3 Significant reduction in intention to consult in future for similar illness (55.3% intervention vs 76.4% control) **p<0.001** CI 0.20 to 0.57	****
Hansen 1990^26^	Reported significant reduction in consultations in intervention group (mean consultations 0.288 (2SD 0.315–0.252) intervention vs 0.426 (0.461–0.390) control group). **p Value not given but states as significant**	**
Herman and Jackson 2010^29^	Significant reduction in choosing to contact HCP first. Pre 69% Post 33% **p<0.0001** Significant reduction in ED (by 58% **p<0.001** 95% CI 0.51 to 0.50) and doctor visits (by 42% **p<0.001** 95% CI 0.33 to 0.46)	***
Isaacman *et al* 1992^27^	Parent reported physician contact showed a non-significant reduction (22.8% control vs 13.2% intervention group). Return to ED rates by day 3 were significantly reduced in intervention groups (3.1% intervention vs 10.1% control group **p=0.05)**	**
Kai 1994^32^	14 parents reported that on 19 occasions Baby Check influenced their decision not to contact a doctor	**
O’Neill Murphy *et al* 2001^30^	High attrition to follow-up resulted in no data on effect on consultation rate	*
Robbins *et al* 2003^12^	Significant reduction in visits to child health clinic (median visits: intervention 4.5 vs control 5 **p=0.039)** No significant difference in GP, HV or minor illness nurse contacts	***
Rosenberg and Pless 1993^21^	Non-significant reduction in ED use in intervention group. Mean total medical visits/year: Control 0.87 (SD 1.5) Intervention 0.7 (SD 1.3)	
Steelman *et al* 1999^22^	No significant differences in clinic or ED use between control and intervention groups, but parents with more than 1 child had significantly more ‘inappropriate’ visits (>1child control group=5 ‘inappropriate’ visits, intervention group=7 such visits vs 1 ‘inappropriate’ visit for both intervention and control in families with 1 child only **p=0.04)**	
Thomson *et al* 1999^33^	No significant difference in total consultations p=0.26, GP p=0.30, out of hours service use p=0.93 or referrals p=0.64	***
Usherwood 1991^35^	No significant difference was found in the number of daytime health centre contacts Significant decrease in home visits in the intervention group for households with one or two children (28% reduction, **p<0.05**) but not for larger families Significant increase in out of hours contacts in the intervention group (mean contacts: 1 child family Control 0.03 vs Intervention 0.10; 2 child C:0.11 vs I:0.23; 3 child C:0.06 vs I:0.30 **p<0.05)**	*
Yoffe *et al* 2011^34^	Statistically significant reduction in ED use in intervention group **p<0.001.** Reductions ranged from 55 to 81% compared to the same month in the previous year	
Summary	6/15 studies significant difference including 1 reduction in intention to consult, 1 reduction in home visits but with increase in out of hours services Quality assessment rating between zero stars (lowest) quality and four stars (highest)	

ED, emergency department; GP, general practitioner; HCP, healthcare professional; HV, health visitor.

#### Knowledge

Nine studies assessed the effect of interventions on parental knowledge of childhood illnesses including fever, upper respiratory infections, febrile convulsion and otitis media (see table 3). Most interventions used multiple methods to provide information, such as written materials supported by verbal explanations (one high-quality study).^12^ ^19^ ^22^ ^23^ ^27^ ^28^ ^36^ Timing of outcome measurement ranged from immediately to 32 months later. Eight studies (one high quality) found a significant increase in parental knowledge after interventions^18^ ^19^ ^22^ ^23^ ^27^ ^28^ ^31^ ^36^ with a spread of 24 h to 12 months for postintervention reassessment. One (high quality) study showed reduction in knowledge at 7 months.^12^

**Table 3:** Effectiveness of interventions on parents’ knowledge

Author (date)	Parent's knowledge (significant results in bold)	Quality
Baker *et al* 2009^18^	Significant reduction in knowledge scores: 54% reduction in responses that fever was dangerous (**p<0.0001**, 95% CI 0.43 to 0.65) 28% reduction in responses that child with fever should be woken (**p<0.0001**, 95% CI 0.19 to 0.39) 30% increase in responses identifying aspirin as inappropriate (**p<0.0001**, 95% CI −0.42 to 0.16)	***
Broome *et al* 2003^19^	Knowledge increased significantly more in both groups than in control group at 24–72 h and 1,3 and 6 months **p<0.03.** No information on the size of the effect provided. Those given individual instruction reported to have higher scores—no p value provided	*
Isaacman *et al* 1992^27^	Parent recall of medication data higher in all groups than other items but with no significant differences between groups. Recall of signs of improvement increased significantly for both interventions groups compared to controls at exit interview, day 1 and 3 (mean correct responses Exit int. Control 0.9, Verbal 25.3, Verbal and Written 56.9; Day 1 C 33.3, V 54.5, V&W 61.0; Day 3 C 44, V 60, V&W 73.2; all **p<0.05**). Recall of worrying signs improved significantly compared to controls at exit and on day 1 (Exit int. C 5.5, V 32, V&W 38.1 ; Day 1 C 19.1, V 37.5, V&W 44.5; Both **p<0.5**). The written and verbal intervention groups performed better than the verbal group at exit interview only for signs of improvement and recall of worrisome signs (**p<0.05**)	**
Kelly *et al* 1996^36^	Indirect measurement of knowledge: No significant difference in level of fever at which antipyretics were administered (p=0.91). A significant difference was found in accuracy of antipyretic dose (n=30 incorrect dose preintervention, 18/30 (60%) accurate doses postintervention **p=0.04**)	**
McCarthy *et al* 1990^23^	Indirect measurement of knowledge: *Reliability of mother's judgements:* intervention group were more likely to agree with clinician than control group: 91.7% vs 72.4% (κ 0.50 vs 0.26) *Specificity of mother's judgements:* Mothers in the intervention group were less likely to score the child's illness as more severe than the paediatrician than those in the control group (Intervention 90% vs 59% control group **p<0.0001**) *Sensitivity of mother's judgements:* Serious illness was the outcome used to measure sensitivity. No difference found between intervention and control group (80% vs 90% respectively)	*
Robbins *et al* 2003^12^	Non-significant reduction in knowledge at 7 months in intervention group	***
Steelman *et al* 1999^22^	Significantly fewer incorrect responses in intervention group at 2 months (Intervention 10.4 vs Control 11.8; **p=0.006**) and at 4 months (Intervention 8.5 vs Control 10.3; **p=0.002**)	
Stockwell *et al* 2010^31^	Significant increase in knowledge/attitude health literacy score (61% **p<0.05**)	**
Wassmer and Hanlon 1999^28^	Significant increase in parental knowledge of febrile convulsion in the intervention group **p<0.05** but these parents children had already had a febrile convulsion. See the original paper for details on size of the effect as these are reported per question asked of parents	
Summary	8/9 showed significant increase in knowledge, although implied in 2 studies and 1 study had high risk of bias. 1 paper showed reduction in knowledge at 7 months. 1 qualitative paper	

#### Anxiety/reassurance

Of the seven randomised controlled studies that reported this outcome (table 4), only one reported significantly reduced concern compared with control group following intervention^26^ (2* quality rating). Using Baby Check to score their baby's illness reassured 41% (14/34)^32^ and 46%^24^ of parents, respectively. In Herman and Jackson's^29^ (high-quality) study the percentage of parents reporting that they were ‘very worried’ when their child was sick reduced by one-third.

**Table 4:** Effectiveness of interventions on parents’ anxiety of reassurance

Author (date)	Anxiety/reassurance (significant results in bold)	Quality
Francis *et al* 2009^25^	No significant difference in level of reassurance	****
Hansen 1990^26^	Significant reduction in worry reported as the main reason for consulting the GP (19% vs 31% **p=0.0075**)	**
Herman and Jackson 2010^29^	Parents reporting being ‘very worried’ when their child is sick reduced by a third (no further statistics available)	***
Kai 1994^32^	11 parents consulted despite low acuity scores to avoid consulting later ‘out of hours’, or because they wanted reassurance Baby Check did not answer their questions or tell them how to manage minor illness	**
Krantz 2001^38^	Parents felt that the fever guide was reassuring and that the decision guide on what to do when was important to include	*
O’Neill Murphy *et al* 2001^30^	At 2 weeks both groups were less anxious. Control 86% Intervention 50%	*
Thornton *et al* 1991^24^	In the first part of the study 46% found using Baby Check reassuring and 4% said it caused anxiety. 6% of mothers reported that Baby Check helped them to decide whether or not to seek advice, 4% were reassured by a low score. Two with high scores were prompted to seek help	****
Summary	1/7 significant reduction in worry. 3 reduced anxiety but descriptive statistics only. 2 qualitative papers	

GP, general practitioner.

#### Satisfaction

Four studies assessed the effects of interventions on parent's satisfaction with their communication with health professionals,^19^ ^25^ and with the educational information received.^27^ ^37^ Two studies reported non-significantly increased satisfaction in control and interventions groups^19^ ^25^ (one high quality), while another reported significantly increased satisfaction for both intervention groups compared to controls^27^ (2* quality). The fourth study suggested a web-based self-triage tool would be well received by parents^37^ (low quality).

#### Confidence

Two of four studies^12^ ^19^ (one high quality) measuring the effect of interventions on parents’ confidence in managing childhood illness at home did not show an increase in levels of confidence. However, Thornton *et al*'s^24^ (high quality) field trials of ‘Baby Check’ found parents’ confidence in the tool itself increased over time, while Kai's^32^ (2* quality) qualitative exploration found that parents felt ‘Baby Check’ had increased their confidence to monitor their child and given them ‘moral support’ for their decision to consult a doctor.

#### Antibiotic prescription

Four studies assessed the effect of interventions on antibiotic prescription. Francis *et al*^25^ (high quality) found a significant reduction in In antibiotic prescriptions given by clinicians in the intervention group (19.5% intervention vs 40.8% control (95% CI 13.7 to 28.9, p<0.001)); and Stockwell *et al*^31^ showed a reduction in the number of parents who sought antibiotics without a prescription or used over the counter medication inappropriately; however this small study (11 parents) failed to report effects on antibiotics sought by parents from health professionals. Two other studies (both high quality)^12^ ^33^ found no significant differences in antibiotic prescribing.

### Factors influencing the effectiveness of an intervention

Factors which may have influenced the effectiveness of interventions were identified from a comparison of study populations and/or the setting of the study and the content, format and delivery of the educational interventions.

#### Content of interventions: range of topics addressed by the interventions

Eleven studies assessed interventions which focused on a single symptom or type of childhood illness alone (such as fever, febrile convulsions, respiratory tract infection, otitis media), while 10 provided information on a range of different childhood illnesses.

Three single-topic studies measured consultation behaviour, of which Francis *et al*^25^ found reduced intention to consult in the intervention compared to control group while two did not.^18^ ^22^ Two single-topic studies assessed anxiety/reassurance, one found no effect^25^ and the other a reduction in intervention and control groups.^30^ Confidence was assessed in one single-topic study^19^ which found no effect. Antibiotic prescribing was assessed in two respiratory focused studies,^25^ ^31^ one of which showed a significant reduction in prescribing in the intervention group in the first 2 weeks postintervention^25^ and the other a non-significant reduction in seeking antibiotics without prescription after the intervention^31^ (only Francis *et al* studied rated as high quality).

Four of the 10 studies evaluating the effects of providing information on multiple childhood illnesses or symptoms showed trends towards reduction in consultation rates or intention to consult^26^ ^29^ ^34^ ^35^ (one high-quality). Four multitopic intervention studies reported a reduction in anxiety or increased reassurance^24^ ^26^ ^29^ ^32^ (one high quality). Confidence improved in two of the ‘Baby Check’ studies^24^ ^32^ (one high quality) but in another (high-quality) study, there was no effect on confidence.^12^ Neither of two high-quality multitopic studies demonstrated a significant reduction in antibiotic prescribing.^12^ ^33^

In summary, reduction in consultation rates, reduction in anxiety and increases in confidence appeared more common in multitopic compared to single-topic interventions, while reduction in antibiotic prescribing was more effective with single illness-focused interventions.

#### Content of interventions: information on assessment and/or management of childhood illness

Four interventions specifically intended to enable parents to assess the severity of their baby's illness and know when to seek medical attention for their child^23^ ^24^ ^32^ ^33^ (two high quality). One of these interventions (a low quality study) informed parents about fever and home management of fever and found that 90% of parents rated the information helpful in decision-making and as a communication tool.^19^ In contrast, nearly one-third of parents did not think the ‘Baby Check’ educational tool was useful,^24^ and a qualitative study of the same tool^32^ revealed that even when parents scored their child's illness as minor they still consulted for the illness within 24 h after the assessment, because they wanted practical advice on management.

#### Content of the interventions: accessibility of the information

Many of the papers provided brief descriptions of the strategies used to make interventions easy to understand for parents. Three (one high quality) designed their interventions specifically for parents with low levels of health literacy.^29^ ^31^ ^38^ The language used in the ‘Baby Check’ score card was simplified to accommodate low health literacy through the translation of professional terms such as ‘reduced tone’ as ‘floppiness’^24^ and a further three studies reported that their interventions were designed for age 11–12-year-old reading level.^30^ ^34^ ^39^ One study specifically mentioned using cartoons and humour to increase the accessibility of information.^34^ There was no identifiable relationship on outcomes between studies which did or did not design interventions for easy reading. However, Krantz's^38^ qualitative study evaluating parents’ views of a fever guide found that parents liked the one page, easy-to-read style, the use of simple diagrams such as a thermometer showing both Fahrenheit and Celsius and pictures of how to measure a child's temperature. Parents felt that these pictures were likely to enhance recall of the information.

#### Delivery method for interventions: interactive or one-way flow

Six studies provided educational interventions to parents in an interactive manner, that is, the parent could engage with the intervention rather than just receiving information:^19^ ^23^ ^25^ ^29–31^ ^36^ two (high-quality studies) showed significant reductions in consultation rates or intention to consult^25^ ^29^ and four significantly improved parental knowledge^19^ ^23^ ^31^ ^36^ (low to 2* quality).

Two additional but low to 2* quality studies^19^ ^26^ used a relatively simple non-discursive method to provide information to parents, showing significant reductions in consultations of up to 88% in a comparison of attendances to an emergency department per month 1 year following the intervention. These shared a common feature: when health professionals gave their booklets to parents, they emphasised that the content was important and would help them to look after their acutely sick child. These findings intimate that educational interventions can be successful even when they are provided using a simple method, but clearly further studies are needed to demonstrate this.

#### Intervention setting

None of the four interventions which were delivered in the waiting room of an emergency department^18^ ^20^ ^21^ ^30^ (one high quality) had significant effects on consultation rates, anxiety or parental knowledge. These studies involved both single topic and multitopic interventions with varying delivery mechanisms and suggest that it is the environment in which the intervention was delivered which is associated with effectiveness, rather than the content of the intervention itself.

Two US studies^29^ ^31^ took place in children's health centres: one high-quality study reduced consultation rates in local emergency departments and primary care^29^ and the other improved parental knowledge.^31^ Peer support and a trustworthy environment were two important factors suggested by the authors as related to this success.

#### Parent involvement in intervention development or evaluation

One high-quality study involved parents in the development^25^ and four in the evaluation of the educational intervention.^19^ ^26^ ^29^ ^35^ Four showed reduction in consultation rates, intention to consult, or improved parental knowledge.^19^ ^25^ ^26^ ^29^ In comparison, studies using existing educational materials as their intervention, without modification and evaluation by its target population, were less successful^12^ ^33^ (both high quality).

## Discussion

This systematic review and synthesis of information resources intending to help parents decide when to seek medical help for an acutely sick child identified measures of effectiveness used to evaluate interventions, as well as factors which appear to influence the effectiveness of interventions. Unlike previous reviews which focused on interventions specifically for respiratory tract infections^40^ or acute paediatric hospital admissions,^41^ our review was broader as we identified factors influencing effectiveness of interventions on parents’ help-seeking behaviour for all common acute illnesses at home.

### Measures of effectiveness

Consultation frequency, knowledge, reassurance/anxiety, satisfaction, confidence and antibiotic prescribing were used as measures of effectiveness. Studies which found reductions in consultation rates^27^ ^29^ ^34^ were all conducted in the USA, which may reflect differences in health service delivery systems and possible financial costs associated with unscheduled consultations. These differences in parental motivations may limit applicability in other countries such as the UK where direct parent-incurred health service costs are less relevant.

Results from studies measuring parents’ knowledge of acute childhood illness indicate that when both verbal and written information were provided, parents were more likely to retain knowledge in the long term than when only given written information.^19^ ^22^ ^23^ ^28^ ^31^ ^33^ ^37^ Verbal reinforcement may signal to parents that health professionals endorse the information.

Providing information did not seem to be directly linked to increased satisfaction, although it is not clear whether the studies we found used a valid measurement tool. Limited information was available about the methods used to measure parent satisfaction, which included a question over the phone,^27^ or using one or two items within a rating scale administered by phone.^19^ ^25^ Satisfaction is a complex phenomenon and it is therefore unlikely that such simple measures will elucidate factors which influence it. No conclusions can be drawn regarding the impact of interventions on parents’ confidence to care for their child.

The effectiveness of interventions at reducing antibiotic prescriptions mirror those of Andrews *et al*'s^40^ review of interventions specifically focused on reducing consultation and antibiotic use in respiratory tract infection, which found that educational materials reduced consultation rates by up to 40%. The two respiratory focused studies which we identified, one from the UK and one from the USA, both indicated a reduction in antibiotic use, while neither of the less focused interventions demonstrated any effect on antibiotic use.

We were unable to easily identify an intervention which works consistently to reduce consultation rates, to improve parents’ knowledge, confidence or satisfaction.

### Factors influencing the effectiveness of an intervention

Interventions providing information on multiple childhood illnesses or symptoms appeared to be more effective (eg, reduction in consultation rates or intention to consult, reduction in anxiety or increased reassurance), compared to interventions addressing single symptoms. This may be because common childhood symptoms, such as fever, cough, sore throat, vomiting and diarrhoea, often occur simultaneously. Therefore, although parents receiving fever education may feel more competent in managing fever, they may continue to seek a medical consultation for other symptoms about which they have less knowledge or confidence. Moreover, educational material which addressed the assessment of illness severity as well as management of minor illness appear to be more effective in supporting parents to care for their children and seek help when necessary: if information is only provided on assessment this may still leave parents needing advice about how to manage, even minor, illness.

Parents’ involvement in the development of educational interventions may improve effectiveness. These findings support the general trend towards involving patients and the public in research,^4^ emphasising the importance of working collaboratively with the end users of interventions.

O'Neill-Murphy *et al*^30^ argued that information provided in an interactive method is more effective in improving knowledge than non-interactive methods. However, our findings do not clearly support this position as we noted significant effects for interventions delivered with, and without, interaction. Involving health professionals in the *distribution* of booklets, with or without an interactive discussion, may increase the perceived value and reliability of the information and motivate parents to read the booklets, trust the home management strategies suggested and, finally, impact on their behaviour. Parents have previously been found to trust information from doctors more than that from other sources.^9^

Studies in the review were conducted in a range of settings; those conducted in emergency departments were the least effective.^18^ ^20^ ^21^ ^30^ Having an acutely sick child is a stressful time for parents, generating considerable anxiety and uncertainty about when to seek medical help.^5^ ^9^ ^11^ Stress can impair learning,^42^ ^43^ therefore it is not surprising that in Chande *et al*'s study only 65% of participants in the intervention group remembered the video in the emergency department. However, two US studies^29^ ^31^ conducted in children's health centres showed reduction in consultation rates in local emergency departments and in primary care^29^ and improved parental knowledge.^31^ We do not know whether interventions delivered in children's centres would similarly work in the UK, although community education on childhood illness has been suggested in a recent UK survey of parents’ first contact choices.^43^

### Strengths and limitations

The strengths of our review lie in its inclusiveness. Given the non-comparable research designs, we used an integrative narrative approach, recognised as an effective method for summarising and synthesising findings across multiple study designs.^16^ ^17^ This approach enabled us to identify influences on effectiveness across a wider range of studies and topics than would have been possible with a single study type or topic focused review. This comprehensive strategy does result in the inclusion of low quality studies whose impact may be questioned and means our recommendations need to confirmed in further studies.

It is possible some studies were missed as the screening of titles and abstracts for inclusion was performed by only one person. The highly heterogeneous nature of the included studies in terms of design, as well as interventions, outcomes measured, populations and settings limited our ability to perform more quantitative syntheses. The literature search was of papers published in English since January 1990. However, it was evident that some of the earlier included studies are already of limited direct relevance to contemporary health services. For example, the ‘Baby Check’ tool used in three studies included a requirement for parents to measure rectal temperature, which is no longer recommended practice. Also no studies compared differing healthcare delivery systems; health systems are likely to have implications on the impact of different interventions.

### Recommendations for clinical practice: how best to provide information to help parents decide when to seek help for an acutely sick child

Our findings indicate that interventions with the following characteristics are more likely to be effective:

- Comprehensive information on childhood illness;
- Information on assessment of children's need for a medical consultation *and* on how to manage minor illness at home;
- Reinforcement or support by local healthcare professionals;
- Delivery away from the stressful environment of the emergency department. This could be in primary care, in the home or in social care settings;
- Coproduction with parents.

Even without the development of new materials for parents of acutely ill children, there are messages here for clinicians using existing materials. Clinicians need to select resources which provide information on multiple common symptoms of childhood illness. Evidence from focus groups parents indicates development with parents is good practice. Interventions in this area can have unexpected consequences which need to be considered prior to implementation, as, for example, one primary care-based intervention which resulted in shifting consultation from day time home visits to the out of hours service.^35^

Information is best provided in primary care or social care settings. Community centres such as SureStart Children's Centres in the UK provide a potential route for the delivery of health information by health professionals, such as health visitors.

### Directions for future research

Most of the studies included in the review were quantitative, providing valuable information on the effects of educational interventions. More qualitative studies are needed, which are able to provide in-depth understanding about what, how and why interventions affect parents’ abilities to assess and manage acute childhood illnesses. This information should be underpinned by research which identifies both parents’ and health professionals’ current use of information resources, and their views on how these resources need to be developed. Finally it is important that any future interventions for parents should be co-developed with parents themselves.^44^ ^45^ Given the rising rates of consultations and the considerable impact this is having on the health service in the UK, as well as on parents, there is a pressing need for larger scale implementation studies taking into account the findings of this review.

## Conclusion

Overall, the majority of reviewed interventions had limited effects on consultation rates. Although many studies showed an improvement in parental knowledge of childhood illness, this did not necessarily lead to more confidence and less anxiety in parents when looking after their child at home. Interventions providing comprehensive information on childhood illness which can be used for both assessing children's need for a medical consultation and for managing minor illness at home were more effective in reducing consultation rates than those focused on a single symptom/illness or only on assessing the child's level of acuity. Interventions also appeared more effective if parents were involved in their development or evaluation.
